# Computer Testing, Formative or Summative, and Proctoring: Does it Matter? Lessons Learned From the Dutch Interuniversity Progress Test of Medicine During the Corona Pandemic

**DOI:** 10.5334/pme.1771

**Published:** 2026-02-11

**Authors:** Jan Hindrik Ravesloot, Jeroen Donkers, Ariadne A. Meiboom, Alexandra M. J. Langers, Bram jacobs, Rene Tio, Cees van der Vleuten, Andreas J. A. Bremers

**Affiliations:** 1 Amsterdam University Medical Center, location Amsterdam Medical Centre, Amsterdam, the Netherlands; 2 Department of Educational Development and Research of the Faculty of Health, Medicine and Life sciences of the University of Maastricht, Maastricht, the Netherlands; 3 Amsterdam University Medical Center, location Free University Medical School, Amsterdam, the Netherlands; 4 Leiden University Medical Center, Leiden, the Netherlands; 5 Department of neurology, University Medical Center, Groningen, the Netherlands; 6 Department of cardiology, Catharinaziekenhuis Eindhoven, Eindhoven, the Netherlands; 7 Department of educational development and research in the faculty of health, medicine and life sciences, Maastricht University, Maastricht, the Netherlands; 8 Radboud University Medical Centre, Nijmegen, the Netherlands

## Abstract

**Introduction:**

The COVID-19 pandemic prompted significant changes in the administration of the Dutch Interuniversity Progress Test of Medicine, offering a unique opportunity to investigate the effects of various test formats. This study explores the impact of transitioning from paper-based to computer-based testing, the shift from summative to formative testing, and the effectiveness of remote proctoring compared to live supervision.

**Methods:**

Data from over 10,000 participants across five medical schools were analyzed.

**Results:**

Results showed no significant difference in student performance between paper-based and computer-based tests. Additionally, remote proctoring proved to be as effective as live supervision in preventing dishonest behavior. Formative testing yielded slightly better results than summative testing in most schools, although the effect varied between institutions.

**Discussion:**

Overall, our study concludes that computer-based testing is a viable alternative to paper-based formats, and remote proctoring can effectively replace live invigilation, at least in our setting and under COVID-19–related circumstances.

## Introduction

The Netherlands hosts eight medical schools, all of which participate in the Dutch Progress Test of Medicine (DPTM) [1]. The DPTM comprises 200 multiple-choice questions, covering major medical disciplines. This national assessment is administered four times per year, with approximately 15,000 medical students taking the test. To accommodate the large number of participants, examinations were typically conducted in large, centralized testing venues such as university halls, municipal halls, or convention centers.

The COVID-19 lockdown in 2020, with strict social distancing measures [2,3], significantly impacted the DPTM, since most exam venues could not accommodate the required number of students. Medical schools adapted by switching to computer-based testing. Four variants were introduced: *(i*) unsupervised at-home tests for students not requiring a summative result, (*ii*) online proctored at-home tests (*iii*) in-facility supervised tests for those needing a summative assessment, and, where feasible, (*iv*) the traditional paper-based tests.

With around 10,000 students participating, we had the unique opportunity to investigate the effects of these three interventions. We examined the impact of switching from paper-based to computer-based testing. Next, we explored the effects of shifting to formative testing. Finally, we compared the online proctored at-home tests to those made under staff-based supervision. We relied on a robust historical dataset to help control for potential biases. Here we report that we observed no significant difference in student performance between paper-based and computer-based tests. Furthermore, remote proctoring proved to be as effective as live supervision in preventing dishonest behavior, at least in our setting, and under COVID-19–related circumstances. Finally, formative testing yielded slightly better results than summative testing in most schools, although the effect varied between institutions. These results may be of interest to teaching staff, practitioners using progress testing and to assessment researchers interested in epistemological implications.

## Materials and methods

### The Dutch interuniversity progress test of medicine (DPTM)

In the Netherlands, medical training consists of a three-year bachelor’s program followed by a three-year master’s program, each with its own examination requirements. Details of the Dutch interuniversity progress test of medicine are summarized elsewhere [1]. In short, the DPTM assesses students on medical knowledge. The required knowledge competencies are specified in the Framework for Undergraduate Medical Education in the Netherlands, which is mandated by Dutch law. The DPTM is administered four times per academic year. Students graduate from medical school after 6 years. The 6 × 4 measurement moments collectively capture students’ longitudinal knowledge growth. These 24 measurement moments represent students’ curricular age, with measurement moment 1 marking the start of the first year and measurement moment 24 marking the end of the sixth year. Each test consists of 200 multiple-choice items, distributed according to a two-dimensional blueprint that balances medical disciplines (e.g., anatomy, pathology, internal medicine) with clinical categories (e.g., cardiovascular, gastrointestinal, nervous system). The multiple-choice questions offer a “question mark” option, allowing students to indicate penalty-free unfamiliarity with the content. Students take the same test simultaneously at all medical schools, ensuring national comparability. After each test, feedback is provided via the PROF (Progress Test Feedback) system, which displays both individual and cohort-level performance trajectories across measurement moments. The design fits formative and summative purposes: it provides ongoing feedback to support self-directed learning while serving as a standardized measure of cumulative medical knowledge. Pass/fail cut-off scores were calculated using the mean and standard deviation across all cohorts, with progressively higher thresholds set from year 1 through year 6. As all students start the bachelor’s curriculum in September, each test has three measurement moments at which bachelor students take the test. A September test for example, will only have the measurement moments 1 (year 1), 5 (year 2) and 9 (year 3). The master’s curriculum has a continuous inflow of students throughout the year and hence a test always includes all 12 measurement moments, i.e., 13–24.

### Adaptations during the COVID-19 regulations

During the COVID-19 pandemic, five of the eight Dutch medical schools used the DPTM and adapted their DPTM test protocols to comply with restrictions. Two major adaptations were implemented. First, the multiple-choice questions were migrated to the Testvision® software platform, enabling computer-based testing. Second, for students not requiring a summative assessment for graduation from either the bachelor’s or master’s program, the tests were primarily formative. Table 1 provides an overview of the adaptations implemented by each medical school. All schools applied formative computer-based, unsupervised progress testing in a domestic setting (medical schools #1-#5, Table 1). The three variants for summative progress testing were as follows. One school (#5) ordered summative computer-based progress testing in a domestic setting with remote online proctoring (Proctorio®). In another three schools (#1–3) only quarantined or isolated students were permitted to take the computer-based test at home with remote online proctoring (Proctorio®). Three schools (#1, #3, #4) used the conventional paper-based test in regular exam facilities. Finally, one school (#2, Table 1) used the computer-based exam for summative progress testing in regular exam facilities. In those instances, the students were supervised directly by staff.

**Table 1:** Distribution of test conditions and student participation across the five medical schools during four assessments in the indicated months in the academic year 2020–2021.


CONDITION/MONTH OF TEST ADMINISTRATION	SEPTEMBER	DECEMBER	FEBRUARY	MAY	TOTAL

**SCHOOL 1**					

Formative Computer-based At-home test	1704	1719	1121	1112	5656

Summative Paper-based Exam venue	80	61	93	122	356

Summative Computer-based Exam venue	0	0	0	0	0

Summative Computer-based At-home test Remote online proctoring	4	1	533	467	1005

total	1788	1781	1747	1701	7017

**SCHOOL 2**					

Formative Computer-based At-home test	1422	1124	1119	1114	4779

Summative Paper-based Exam venue	0	0	0	0	0

Summative Computer-based Exam venue	283	69	75	516	943

Summative Computer-based At-home test Remote online proctoring	12	536	528	25	1101

total	1717	1729	1722	1655	6823

**SCHOOL 3**					

Formative Computer-based At-home test	1683	1655	1454	1666	6458

Summative Paper-based Exam venue	285	276	420	196	1177

Summative Computer-based Exam venue	0	0	0	0	0

Summative Computer-based At-home test Remote online proctoring	29	25	31	4	89

total	1997	1956	1905	1866	7724

**SCHOOL 4**					

Formative Computer-based At-home test	1759	584	875	586	3804

Condition/Month of test administration	September	December	February	May	Total

Summative Paper-based Exam venue	168	1287	952	1212	3619

Summative Computer-based Exam venue	0	0	0	0	0

Summative Computer-based At-home test Remote online proctoring	0	0	0	0	0

total	1927	1871	1827	1798	7423

**SCHOOL 5**					

Formative Computer-based At-home test	855	676	639	624	2794

Summative Paper-based Exam venue	0	0	0	0	0

Summative Computer-based Exam venue	0	0	0	0	0

Summative Computer-based At-home test Remote online proctoring	114	327	705	712	1858

total	969	1003	1344	1336	4652

**TOTAL**					

Formative Computer-based At-home test	7423	5758	5208	5102	23491

Summative Paper-based Exam venue	533	1624	1465	1530	5152

Summative Computer-based Exam venue	283	69	75	516	943

Summative Computer-based Remote online proctoring	159	889	1797	1208	4053

total	8398	8340	8545	8356	33639


All students were allotted four hours to complete the exam. The computer-based tests were organized into four blocks of 50 items presented in random order. Once a block was completed, it could no longer be accessed, and proctoring was briefly paused. This structure allowed students, engaged in summative assessments, to address any physical needs during the exam without compromising test security.

### Data acquisition and outcomes

The time spent by students on the computer-based tests was derived from the server data, categorized by curricular age, and test conditions. These results were then compared with historical data from paper-based tests.

Pass-fail and pass-good cut-off scores were determined using a relative standard method and based on the summative tests only: the mean score minus one standard deviation for pass-fail and plus 0.5 standard deviation for pass-good decisions. Comparisons were made across the entire group and historic data from the previous five years was used as a benchmark to detect any effect of the exceptional circumstances on the relative standard.

For each student, a pre-COVID expected score was calculated based on previous test results and individualized growth trajectories, drawn from historical progress test data. To evaluate potential differences in test duration that could indicate whether students made unauthorized use of external resources during formative assessments, the completion times for summative and formative settings were compared. Even in formative assessments consulting external sources for answers violates examination regulations and undermines the fundamental purpose of progress testing.

To detect any potential anomalies, z-scores were calculated per student. A z-score (or standard score) is a statistical measure that quantifies how far and in what direction a data point deviates from the mean, measured in fractions of standard deviations. Students with z-scores exceeding 3.5 were excluded from the analysis, and the evaluation was repeated to ensure accuracy. Score distributions, broken down by measurement moment (curricular age), were analyzed across different test conditions, including both formative and summative formats, as well as z-scores relative to each student’s average score from the four tests administered in the previous year.

Each participating school’s scores, again segmented by the measurements moments, were also compared to their own historical data. The average z-score for each school was then calculated and cross-referenced against data from the past five years to assess any variations. Additionally, summative test scores were evaluated in relation to historical data for each institution, with z-scores used to identify any specific impacts of formative testing across different student groups.

### Statistical methods

All analyses and figures were created using R [4].

To evaluate the difference in test length between paper-based and computer-based admissions, we applied a censored Cox-regression. The cutoff point for censoring was 240 minutes. The model included measurement moment (mm) and medical school as covariate and the admission condition as an independent variable (model formula: Surv(length, uncens) ~ mm + condition + school).

To test the effects on the results of the progress test for formative or summative admissions, we applied a linear mixed-effect model with the z-score as dependent variable, the offset of student z-scores, and the slope on test-number as random effects and mode (formative vs. summative), test-number, measurement moment and university as independent variables, with an interaction between university and mode (model formula: zscore ~ testnr + mm + school * mode + (1+testnr|studentid)). For each effect, t-tests were performed using Satterthwaite’s method, which implicitly corrects for multiple testing. Significance was accepted at p < 0.05. We applied a bootstrap method to estimate 95% confidence intervals (CIs) for the effects.

For the difference in score between paper-based and computer-based admissions, we restricted the data to summative assessments only. First, we only used data from the COVID period and fitted a mixed-effect model using measurement moment and school as covariates and only the offset as random effect (model formula: zscore~ mm + school + medium + (1|studentid)). Next, in order to further investigate the effect of computer-based admissions and correct for individual student growth, we included results from all paper-based admissions from the period 2015–2020 and extended the model to include a random slope. (model formula: zscore~ mm + testnr + school + medium + (1+testnr|studentid)). In these models we also used Satterthwaite’s method to compute p-values per effect and bootstrapping to estimate CIs.

These analyses were performed in R using LME4 [5], LME4 refers to the R package “Linear Mixed-Effects Models using ‘Eigen’ and S4”, a widely used tool for fitting mixed-effects models. The correlations between ‘medical school’, ‘test mode’ and student scores were visualized using the R package “effects” [6].

We used the combined data of academic years 2015–2021 as a benchmark to measure the effect of the test variant (summative vs. formative). We corrected for possible effects of time, taking into consideration the variability between the tests that were delivered over a six year time span. Furthermore, we corrected for differences in student aptitudes. This left the medical school and the interaction between the medical school and the test format as the remaining variables that could potentially confound the results.

## Results

The number of students that took the four tests during the COVID-19 lockdown, in September and December 2020 and February and May 2021, ranged from 8340 to 8545 per test (Table 1). The data pertaining to these students are presented in this report.

### From Paper-Based to Computer-Based Progress Testing: Effect on Test Completion Time

**Figure 1:**
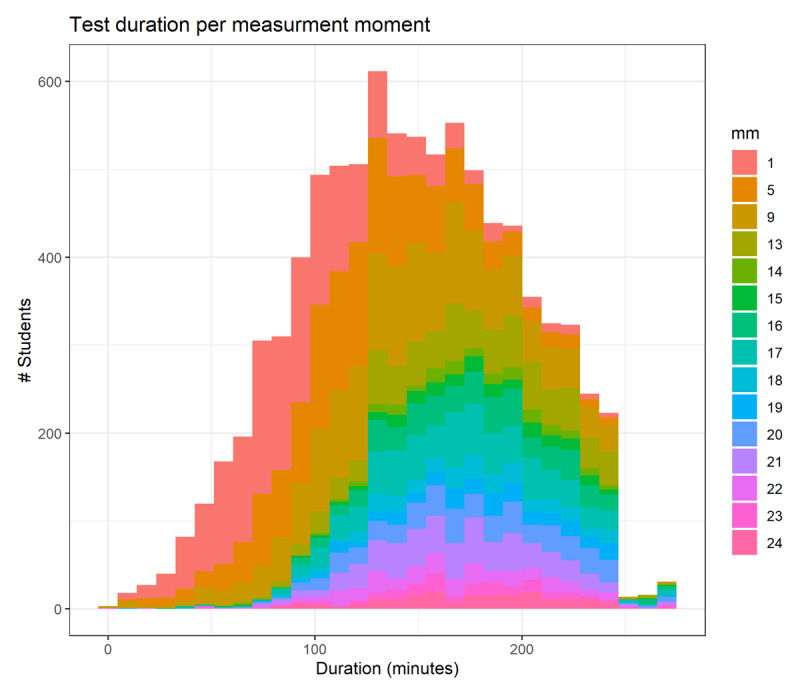
Time spent on the September computer-based test. In the bachelor’s curriculum, age groups are synchronized (1, 5, and 9), while in the master’s curriculum, all curricular age groups are represented (13–24). Y-axis: number of students; X-axis: time spent on the progress test (in minutes). Curricular age groups are distinguished by colour coding. A small number of students with physical or mental challenges were granted additional time beyond the standard 240-minute limit by the boards of examiners of their medical school. See text for further details.

Figure 1 summarizes the time spent on the computer test. The duration data showed a right-skewed distribution. The test completion time gradually increases with curricular age. This pattern is highly comparable to the paper-based tests in the pre-COVID era (data not shown).

**Figure 2:**
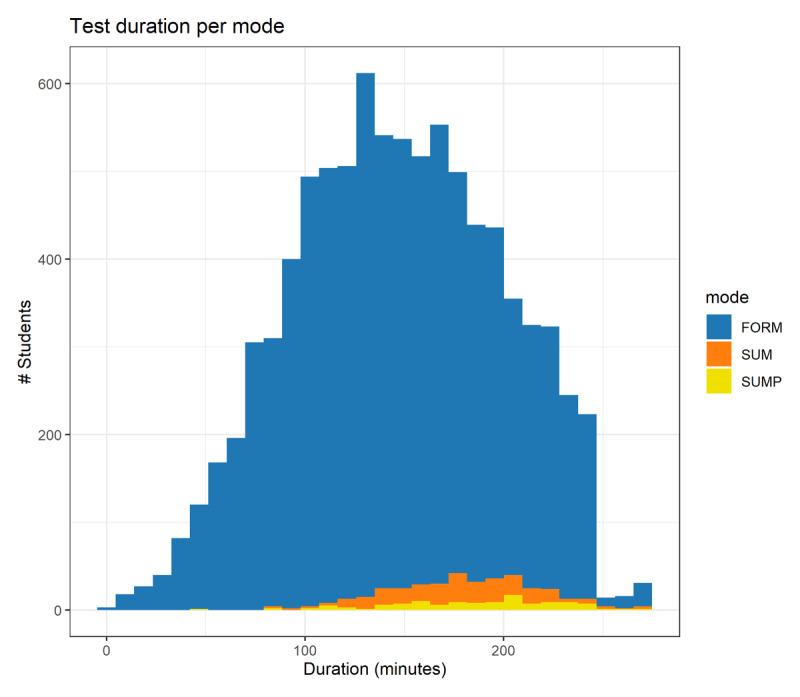
Time spent on formative assessments (FORM, in blue, 9371 students), summative computer-based assessments (SUM in orange, 300 students), and summative paper-based assessments (SUMP in yellow, 163 students), all from curricular age group 1. Y-axis: number of students; X-axis: time spent on the progress test (in minutes). Extended time was granted for a small number of students with physical or mental challenges by the boards of examiners of their medical school. See text for additional details.

Figure 2 summarizes the time spent on formative and summative tests for curricular age group 1. Summative tests, both on paper and computer, took more time than formative tests. The non-negative, right-skewed nature of the duration data justified the use of a Cox regression model. Its interpretable parameters facilitated parametric comparison across the different testing conditions. The Cox parameters of both summative tests, 0.80 (95%CI = [0.68, 0.94]) and 0.67 (95%CI = [0.54, 0.84]]) for the paper-based and computer-based test respectively, were significantly different from the one computed for the formative test indicating that the time spent on the summative tests was higher, p < 0.006.

### From Paper-Based to Computer-Based Progress Testing: Effect on Student Scores

Next, we investigated the question whether the scores of the paper-based test differed from the computer-based test. We observed a slightly but significantly better performance on the computer-based test. In terms of z-scores (effect size) the results on the computer-based test were on average 0.09 SD better (95%CI = [0.003, 0.176], p < 0.05, n = 10098). When we corrected for heterogeneity in the ways each school dealt with the Covid lockdown (Table 1), by including historical data as far back as 2015, the effect size increased to 0.11 SD (95%CI = [0.081, 0.136], p < 0.001, n = 83603). In terms of actual scores this amounts to 1–5 points on a 100-point scale, depending on the measurement moment. The time span, from 2015 onwards, was chosen in order to have all previous results on all students up to the sixth-year students.

### From staff-based supervision to Proctorio® – based supervision

We addressed the question of whether the scores of the Proctorio® supervised computer-based tests differed from the computer-based tests supervised by staff. We observed no significant difference (n = 4968).

### From summative to formative progress testing

**Figure 3:**
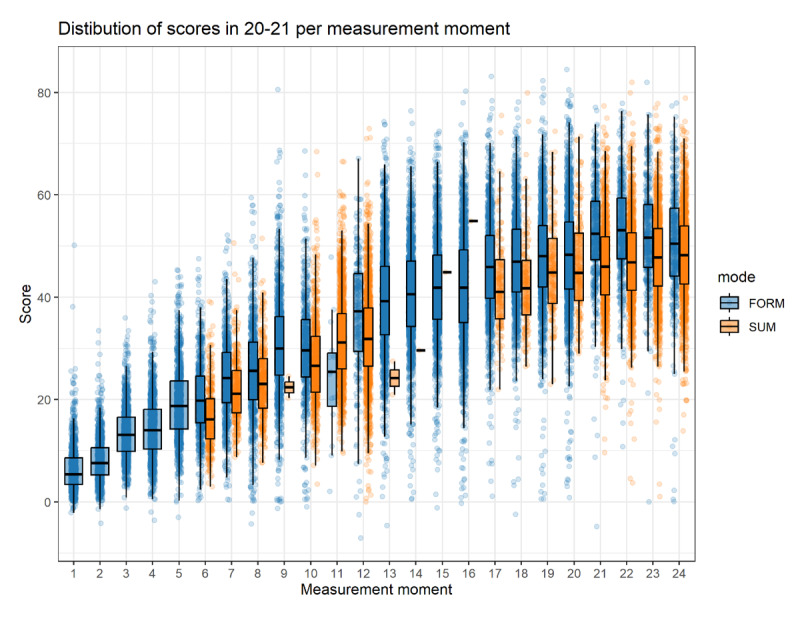
Distribution of individual student scores per curricular age group on formative (FORM blue) and summative (SUM, orange) progress tests. All scores of all tests between in academic year 2020–2021 of 5 universities are included. Their z scores were computed and the outliers (z > 3 and z < –3) removed. Every dot represents one student. Superimposed box-plots summarize the data. The median value is indicated as well as the first and third quartile and the 5th and 95th percentile. See text for details.

Figure 3 shows individual students’ scores per measurement moment (curricular age) group on formative and summative progress tests. Across all measurement moments the scores go up for both formative and summative tests. They level off around measurement moment 15 and higher. Students generally scored higher on formative tests compared to summative tests. The distributions for formative scores are slightly shifted upward, with higher medians and narrower interquartile ranges, suggesting more consistent performance. In contrast, the summative test distributions tend to show greater variability and lower median z-scores, indicating that students performed less uniformly and, on average, slightly worse.

**Figure 4:**
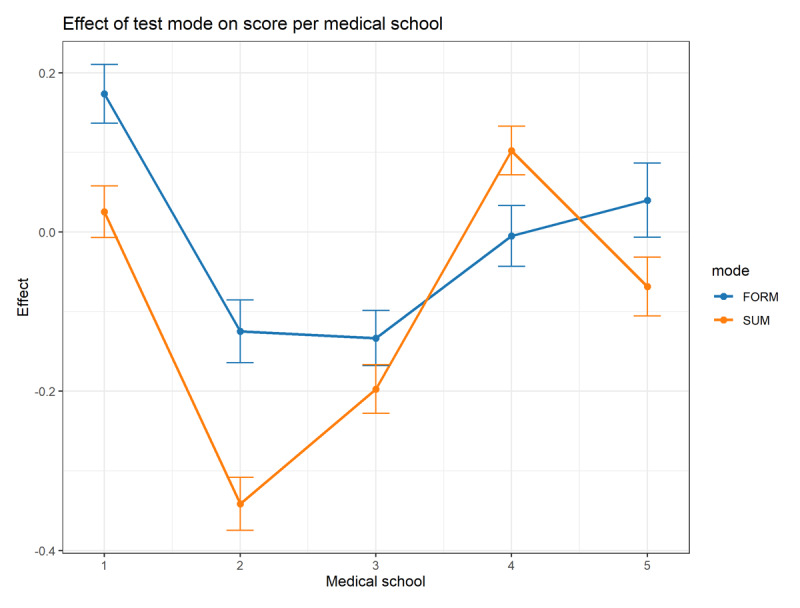
Pooled z-scores of summative (orange) and formative tests (blue) of 106851test results obtained between 2015 and 2021. Formative results of university 1 are used as a reference for comparisons. There is a significant interaction between university and the effect of the test variant.

We applied a correlation analysis and we corrected for the differences in average performance level between the various medical schools. The graphical outcome of the analysis is summarized in Figure 4. The correlation parameters ‘medical school’ and ‘test format’ (formative vs. summative) were significantly different between the institutions. We found that the medical school was a strong predictor of whether a student performed better or worse on the formative variant. At medical schools #1, #2, and #3, students performed significantly better on the formative variant, while at medical school #4, students performed better on the summative variant. The difference observed at medical school #5 did not reach statistical significance. Follow-up analysis revealed that fixed effects, which account for characteristics constant across observations, had minimal impact on the scores. This means that factors that are constant across all students, such as fixed institutional characteristics (e.g., curriculum design, teaching methods), had little influence on the differences in students’ scores. In contrast, random effects, which capture variability that is not explained by these fixed characteristics, such as individual differences among students or variations between groups that were not directly measured, accounted for over 60% of the variance (data not shown). We ruled out the possibility that medical schools allowed only their top-performing students to sit the formative tests. And because the time spent on the summative test was higher, this makes is unlikely that in the formative, unsupervised, setting, time was used to consult external resources. The interaction between medical school and progress test format was shown to be both substantial and variable.

## Discussion

We examined the impact of switching from paper-based to computer-based testing and the effects of shifting to formative testing. We also compared online proctored at-home tests to those made under staff-based supervision.

### Computer-based testing versus paper-based testing

In our analysis, the scores using computer-based tests were slightly higher than on paper. This difference, however small, was statistically significant. Interestingly, Karay et al. found comparable results when looking at the average performing students. However, they found ‘significant differences in processing time between high-performing students and differences in guessing behavior between low-performing students on the paper-based versus computer-based test’ [8]. Since they found these differences between high and low performing students, they concluded that ‘it is still unclear whether computer-based tests influence students’ test scores’. Several studies investigated the difference between paper and computer-based tests, in social, linguistic and science/mathematical subjects, some of which demonstrate better results with paper-based tests [9,10] others show no difference [11,12,13]. The DPTM spans a great variety of subjects. Gordanier et al. demonstrated, for physical and mathematical sciences, an advantage of paper tests, increasing with the complexity of the subject [14].

Although previous reports found computer reading to be slower and less accurate [15], more recent data suggest less reading time for computer-based testing [8]. Getting used to digital testing might take more than a year [9]. In our setting, time spent on the computer test was not longer than paper-based. These differences may be explained by the fact that the technology of the screens has improved and students now are more accustomed to reading from screens than they were 20–30 years ago.

### Formative vs. summative

We observed that students spent the most time on summative tests. At least part of this difference could be explained by the fact that higher curricular age students, in particular at the end of the bachelor and the end of the master, were overrepresented in the summative group; the time spent on the test gradually increases with curricular age as the number of subject items that the student has gained knowledge of, grows. Moreover, students at the end of the bachelor or master may need to just pass this one test to obtain their degree.

We found that the medical school strongly predicted whether a student performed better or worse on the formative variant. Most of the variance was explained by random effects rather than fixed effects. This finding aligns with four empirical studies in United Kingdom medical education, which consistently show that individual-level variability accounts for most of the performance variance. Institutional characteristics contribute only a modest additional proportion. For example, within a single fixed institutional Problem Based Learning curriculum, student-level formative assessment performance strongly predicts OSCE and progress test outcomes, indicating that individual variability rather than institutional characteristics drives most observed score differences [16]. Because formative assessment was mandatory and identical for all students, with the same format, timing, and grading weight, institutional exposure is effectively controlled. As a result, between-student differences in performance cannot be attributed to differential access or participation but instead reflect how individual students respond to the same conditions (e.g., preparation, engagement, motivation). This supports the interpretation that the majority of performance variance is driven by individual-level factors rather than institutional structure. Secondly, a nationwide analysis, examining the volume and intensity of summative assessment, found moderate correlations with postgraduate examination performance after controlling for entry qualifications, but substantial unexplained variance remained attributable to non-institutional factors. A correlation of ~0.5 indicates that more than half of the total variance in postgraduate performance is not explained by institutional assessment policy alone [17]. A third study, on Membership of the Royal Colleges of Physicians exam outcomes, showed that, although between-school differences existed, approximately 62% of the variance was explained by individual pre-admission qualifications, with only a modest additional contribution from school-level educational factors after controlling for selection effects [18]. Finally, a multi-institutional mixed-effects study of formative virtual patient use and self-assessment found that individual engagement and formative performance were the primary drivers of summative outcomes, while institutional clustering explained only a limited proportion of variance. Although students were nested within 20 medical schools, formative performance and self-assessment together explained only 26% of total examination variance, with most remaining variance attributed to unmeasured individual factors such as preparedness and motivation [19]. Overall, while differences between schools exist, individual student factors explain most of the performance variation, with institutional structure playing a secondary role.

We thus propose that medical school educational policies play an important role in priming and preparing the students if difficult circumstances demand a change of attitude towards learning and testing, thereby influencing the individual student factors that control test performance. In addition, absence or presence of continued teaching during the lockdown or information to students about the value of formative tests, may also have played a role. Even though we could exclude a number of obvious confounders, consultation of peers or resources in the unsupervised formative groups as an explanation for the better results in this group can never be excluded with certainty, though a prolonged testing time might be expected in such cases. Another hypothesis could be that students in the formative setting would be less concerned with a penalty for wrong answers [7].

### Remote proctoring versus direct supervision

No significant difference was observed in the computer-administered test, whether supervised directly or by remote proctoring. Direct supervision is a system that matured to a stage where fraud is reduced to an acceptable minimum. Remote proctoring was new, however, to most students. Alessio et al. demonstrated that students using remote proctoring scored lower and spent less time on their tests compared to students who did online tests without proctoring [20]. The remote proctoring system Proctorio® was demonstrated by Bergmans et al. to be easily cheated on, as it shows good specificity but very low sensitivity for fraud in a setting of students of a technical university. The efficacy of remote proctoring seems to be based on the deterrent effect, caused by the awareness that the system is present, rather than its potential to detect fraud [21]. Therefore, it is probably just a matter of time until the student population becomes aware of the possibilities to evade these systems; on the other hand, refinement and technological improvements may maintain the effect of remote proctoring. Besides, ethical considerations should not be ignored [22].

### Limitations

Due to the COVID pandemic’s acute onset, the various ways of administering the test had to be organized in line with the national regulations on COVID restrictions at very short notice. The facilities that were locally available dictated in which way students took their test. Corrections for bias relied on historic data. Due to the vast numbers of participating students and several years of historic data, these corrections are robust.

The majority of the students in this study had a history in which they were accustomed to the DPTM as a summative test. It is not proven that their attitude to the formative DPTM is identical to future students for whom the progress test was a formative exercise from the beginning.

## Conclusion

These results show, for the DPTM, that computer-based testing is interchangeable with paper-based tests. Computer-based summative vs. formative results were comparable. In our cohorts, remote proctoring appeared effective, but it is unclear how, or whether, students will learn to evade this system. Though students spent more time on summative tests compared to formative ones, results were alike, suggesting that formative tests may allow a reliable estimate of ability of students. We propose that medical school educational policies, shaped by unanticipated circumstances like the COVID-19 lockdown, influence students’ study behaviors and attitudes toward examinations.

Overall, the study concludes that computer-based testing is a viable alternative to paper-based formats, and remote proctoring can effectively replace live invigilation.
